# Cerebral infarction following bee stings: Case report and literature review

**DOI:** 10.1515/tnsci-2022-0225

**Published:** 2022-07-07

**Authors:** Shuiquan Yang, Jack Wellington, Juanmei Chen, Robert W. Regenhardt, Alex Y. Chen, Guilan Li, Zile Yan, Pingzhong Fu, Zhaohui Hu, Yimin Chen

**Affiliations:** Department of Neurology and National Advanced Stroke Center, Key Discipline of Traditional Chinese Medicine of Guangdong Province, Foshan Sanshui District People’s Hospital, Foshan, Guangdong Province, China; School of Medicine, Cardiff University, Wales, United Kingdom; The Second Clinical College, Guangzhou Medical University, Guangzhou, China; Department of Neurology, Massachusetts General Hospital, Harvard Medical School, Boston, Massachusetts, United States of America; Department of Neurology, University Hospital, Case Western Reserve University, Cleveland, United States of America; Department of Radiology, Foshan Sanshui District People’s Hospital, Foshan, Guangdong Province, China; Medical Department and National Advanced Stroke Center, Key Discipline of Traditional Chinese Medicine of Guangdong Province, Foshan Sanshui District People’s Hospital, Foshan, Guangdong Province, China

**Keywords:** bee stings, cerebral infarction, thrombectomy, case report

## Abstract

**Background:**

To date, only 25 cases of cerebral infarction following a bee or wasp sting have been reported. Due to its rarity, undefined pathogenesis, and unique clinical features, we report a case of a 62-year-old man with progressive cerebral infarction following bee stings, possibly related to vasospasm. Furthermore, we review relevant literature on stroke following bee or wasp stings.

**Case presentation:**

A 62-year-old retired male presented with progressive ischemic stroke after bee stings to the ear and face. Initial magnetic resonance imaging of the brain showed small punctate infarcts in the left medulla oblongata. Head and neck computed tomography angiography showed significant stenosis in the basilar artery and occlusion in the left V4 vertebral artery. The patient received intravenous alteplase (0.9 mg/kg) without symptomatic improvement. Digital subtraction angiography later demonstrated additional near occlusion in the left posterior cerebral artery (PCA). Thrombectomy was considered initially but was aborted due to hemodynamic instability. Repeated CT brain after 24 h showed acute infarcts in the left parieto-occipital region and left thalamus. The near occluded PCA was found to be patent again on magnetic resonance angiography (MRA) 25 days later. This reversibility suggests that vasospasm may have been the underlying mechanism. Unfortunately, the patient had persistent significant neurological deficits after rehabilitation one year later.

**Conclusion:**

Cerebral infarction following bee stings is rare. There are several proposed pathophysiological mechanisms. While the natural course of this phenomenon is not well characterized, early diagnosis and treatment are essential. Furthermore, it is important to establish standardized care procedures for this unique entity.

## 1 Introduction

Wasp or bee stings are common around the world. Typical allergic symptoms include mild urticaria to severe anaphylaxis. Various unusual reactions after bee stings have been reported, involving neurological, renal, cardiac, pulmonary, and ocular systems [1]. To date, only 25 cases of cerebral infarction following a bee or wasp sting have been reported since the initial index case in 1962. Here, we report a case of a 62-year-old man with progressive cerebral infarction following bee stings and review relevant literature (Table 1) [2,3,4,5,6,7,8,9,10,11,12,13,14,15,16,17,18,19,20,21,22,23,24,25,26]. This rare phenomenon has unclear pathogenesis and clinical features, and there is no consensus on management.

**Table 1:** Case reports of cerebral infarction following bee or wasp sting

Author	Region/Year	Age/Sex	Wasp/Bee	Clinical features	CT/MR findings	Onset time	Angiography	Treatment	Prognosis
Day et al. [2]	US/1962	36/M	Wasp: neck, face, and arms	Headache, hemiplegia, seizure, coma	Necropsy: left hemorrhagic cortical infarction; pontine infarction	15 min	NR	Anti-allergic phenobarbital	Died
Romano et al. [3]	US/1989	1.4/M	Wasp: inner upper lip	Hemiparesis, facial weakness	Left putamen and caudate infarctions	4 days	Left ICA occlusion	Anti-allergic	Full recovery
Riggs et al. [4]	US/1993	38 y/M	Wasp: multiple; face and neck	Hemiplegia, aphasia	Left MCA infarction	2 days	Left ICA occlusion	Unrecorded	NR
Riggs et al. [5]	US/1994	52/M	Wasp	Dysarthria, hemiparesis, quadriparesis	Left parietal and insular cortical infarctions	A few hours	Right ICA occlusion Left ICA near-complete occlusion	Anti-allergic	NR
Crawley et al. [6]	US/1994	30/F	Wasp: arm	Visual deficits, hypotension	Left occipital infarction	45 min	NR	Anti-allergic	Full recovery
Bhat et al. [7]	India/2002	30/M	Bee: multiple; all over body	Dysarthria, vertigo, tinnitus, and bilateral cerebellar signs	Bilateral cerebellar hemorrhagic infarction	<1 day	NR	Anti-allergic reduced intracranial pressure	Died
Sachdev et al. [8]	India/2002	40/M	Wasp: face	Left hemiplegia, slurred speech	Right ventral pons and right cerebellum infarctions	10 h	NR	Reduce cerebral edema aspirin	Improved
De-Meing Chen et al. [9]	Taiwan/2004	71/F	Wasp: head, face, and limbs	Facial palsy, paraplegia	Right MCA territory infarction	1 day	Occlusion of the infrarenal aorta	Thrombectomy, anticoagulant plasmapheresis	The patient received rehabilitation programs and was discharged on the 56th day
Schiffman et al. [10]	US/2004	57/F	Bee: neck, head, eye, face, arm	Left homonymous hemianopia	Large right temporo-occipital hemorrhagic infarction	2 days	Right PCA P1 occlusion	Anti-allergic antiemetics	Improved
Taurin et al. [11]	French/2006	36/M	Wasp: location NR	Vomiting, syncope	Left dorsal medulla infarction	14 days	NR	Anti-allergic	Full recovery
Temizoz et al. [12]	Turkey/2009	60/M	Bee; head, face, limbs	Hemiplegia, dysarthria	Bilateral frontal lobe infarcts, right temporoparietal and bilateral centrum	2 h	NR	Anti-allergic and aspirin	Improved slight left hemiparesis
Vidhate et al. [13]	India/2010	8/M	Wasp: eyebrow nasal bridge	Hemiplegia， altered sensorium	Infarcts in left frontoparietal region, right subcortical area, and posterior limb of the left internal capsule	8 days	CTA normal	Systemic antibiotics, anticoagulants	Improved right-sided complete ophthalmoplegia
Dechyapirom et al. [14]	US/2011	64/M	Bee: face, neck, chest extremities	Hemiplegia, facial palsy, chest pain	Large right MCA territory infarction	16 h	NR	Anti-allergic rt-PA	Recovery
Rajendiran et al. [15]	India/2012	25/M	Bee: head and neck	Vomiting, monoplegia, transient visual loss	Right frontoparieto-occipital infarct with hemorrhagic transformation	1 day	NR	Anti-allergic antiemetics	Full recovery
Viswanathan et al. [16]	India/2012	59/M	Bee: face, neck, scalp, chest	Disorientation, dysarthria, facial palsy, hemiplegia, seizures,	Right perisylvian, peri-insular, and parietal cortices infarct	2.5 h	NR	Anti-allergic aspirin, atorvastatin, and heparin	Improved
Jain et al. [17]	India/2012	70/M	Bee	Altered sensorium, hemiplegia	Left frontalparietooccipital infarction， lacunar infarcts of bilateral gangliocapsular	6 h	MRA normal	Anti-allergic	Improved
Bilir et al. [18]	Turkey/2013	35 M	Bee: multiple; NR	Change in consciousness, dyspnea, hemiparesis	Left MCA infarction	6 h	Neck MRA normal	Antiallergic	Residual right hemiparesis
Wani et al. [19]	India2014	40/M	Wasp: multiple; face, head, and neck	Deterioration in consciousness, hemiplegia, obtundation	Left thalamic, left parietooccipital, bilateral cerebellar hemispheres, and pontine infarction	1 day	NR	Anti-allergic	vegetative state
An et al. [20]	Korea/2014	50/M	Bee	Left involuntary movements	Right temporal infarction	27 h	Right M2 of MCA occlusion	Anti-allergic haloperidol aspirin	Recovery
Kulhari et al. [21]	US/2016	44/M	Wasp: leg	Hemiparesis, facial palsy, dysarthria	Multiple infarctions in right MCA	1 h	Vasoconstriction in the bilateral proximal MCA arteries	Anti-allergic rt-PA	Recovery
Guzel et al. [22]	Turkey/2016	59/M	Bee	Mild shortness of breath left hemiplegia,	Right frontotemporoparietal infarction	A few hours	NR	Anti-allergic	Died
Dalugama and Gawarammana [23]	Sri Lanka/2018	69/F	Wasp:	Hemiplegia, aphasia	Left posterior frontal white matter infarction	NR	NR	Aspirin and atorvastatin	Improved
Gupta et al. [24]	India/2019	41/F	Honeybee: arm	Seizure, hemiparesis, dysarthria, unconscious	Bilateral thalami, left frontotemporoparietal infarctions， hemorrhage transformation	3 h	NR	Antiepileptics,	Died
Elavarasi et al. [25]	India/2020	41/M	Bee	Hemiparesis, dysarthria	Massive right MCA territory infarction	5 h	NR	Antiepileptics heparin, antiplatelets hemicraniectomy	Died
Ramlackhansingh and Seecheran [26]	Trinidad and Tobago/2020	70/M	Africanised honey bee: face, forearms, shoulders, and back	Dysphasia, hemiparesis	Left parietal lobe and left basal ganglia infarctions	1 day	Normal	Antiallergic aspirin	Full recovery
Current study	China/2021	62/M	Honey bee face, neck	Speech disorder, hemiparesis	Left parieto-occipital lobe, basal ganglia, thalamus infarctions	2 h	Stenosis of bilateral VA and BA Occlusion of P1 segment of right PCA	Anti-allergic rt-PA	Sequela

**Abbreviations:** BA, basilar artery; CT, computerized tomography; CTA, computerized tomography angiography; DIC, disseminated intravascular coagulation; DSA, digital subtraction angiography; ECG, electrocardiogram; F, female; ICA, internal carotid artery; M, male; MCA, middle cerebral artery; MRA, magnetic resonance angiography; MRI, magnetic resonance imaging; NIHSS, National Institute of Health Stroke Scale; NR, not reported; PCA, posterior cerebral artery; rt-PA, recombinant tissue plasminogen activator; VA, vertebral artery.

## 2 Case presentation

A 62-year-old retired male with only mild hypertension was brought to the emergency department 1 h after bee stings to the ear and face. The initial symptoms were nausea, vomiting, and headache. On examination, he was found to have dysarthria, bulbar weakness, and right hemiparesis involving the arm and leg. The National Institutes of Health Stroke Scale score was 5. His blood pressure on presentation was 120/77 mmHg. The electrocardiogram showed sinus rhythm with a heart rate of 72 beats per minute. His initial non-contrast head CT was negative for hemorrhage or any acute processes. To treat a possible allergic reaction, he was given intravenous methylprednisolone and calcium gluconate. Given the possibility of acute stroke, intravenous alteplase (0.9 mg/kg) was also administered. Unfortunately, his symptoms did not improve.

**Figure 1:**
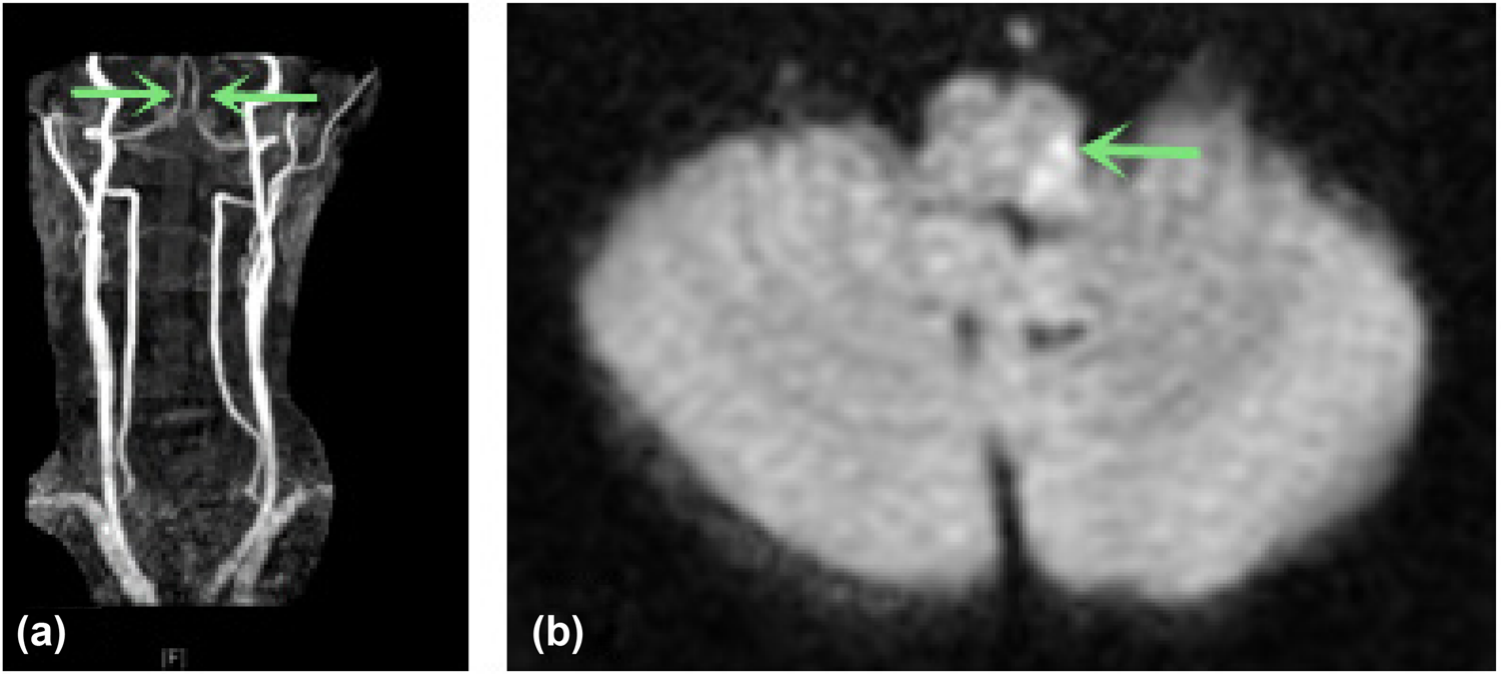
(a) Patent bilateral vertebral arteries (arrow) on CTA neck from three years prior to presentation; (b) admission head MRI (143 min after symptom onset) which showed small punctate infarction in the left medulla oblongata (arrow).

Magnetic resonance imaging (MRI) of the brain was performed, which showed acute infarcts in the left medulla oblongata (Figure 1).

**Figure 2:**
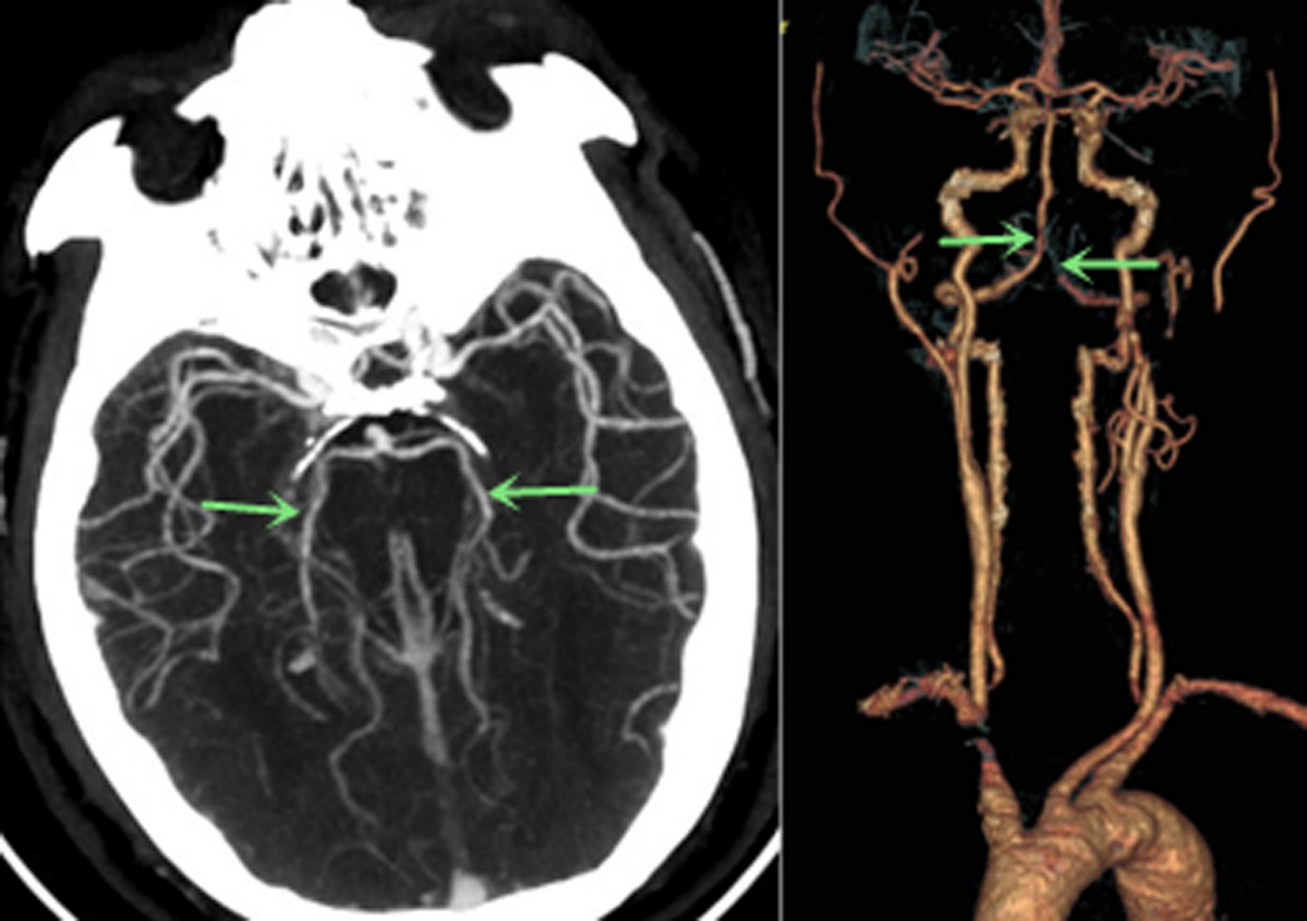
Admission CTA (225 min after symptom onset) showed significant stenosis in the basilar artery, and occlusion of the left V4 segment of the vertebral artery(arrow). Both bilateral posterior cerebral arteries were patent at this time (arrow).

**Figure 3:**
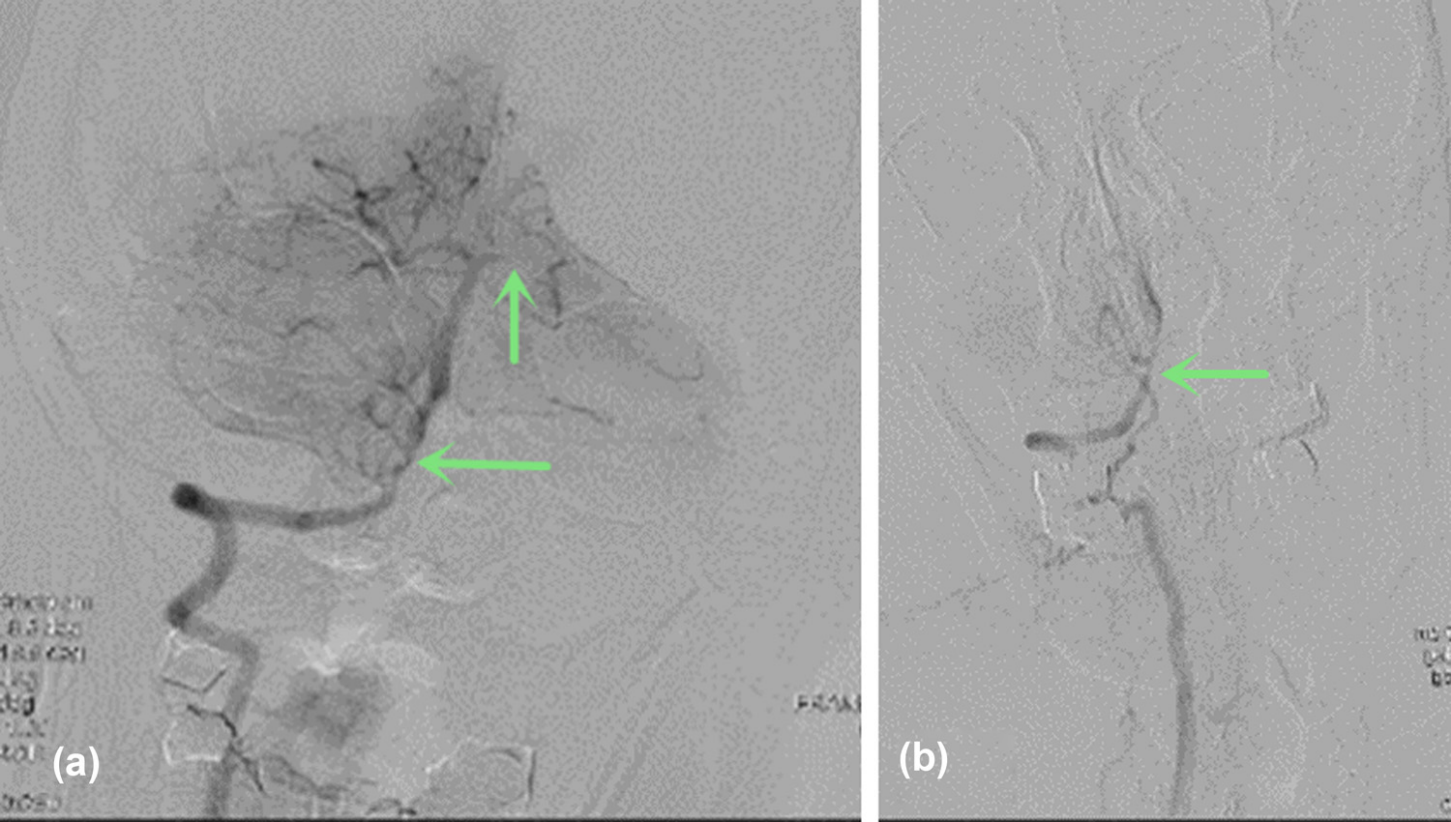
DSA (8 h after symptom onset) showed (a) significant stenosis in the right V4 segment of the vertebral artery and occlusion in the left PCA (arrow); (b) occlusion in the left V4 segment of the vertebral artery (arrow).

**Figure 4:**
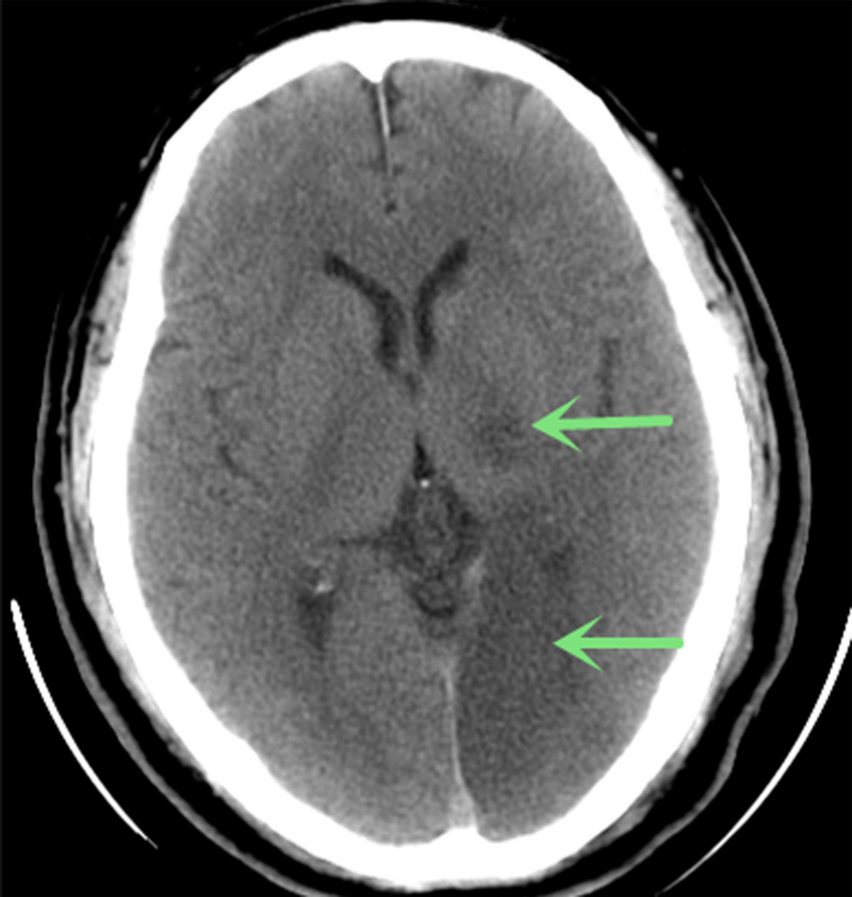
Head CT (18 h after symptom onset) showed ischemic infarcts in the left parieto-occipital lobe and thalamus (arrow).

**Figure 5:**
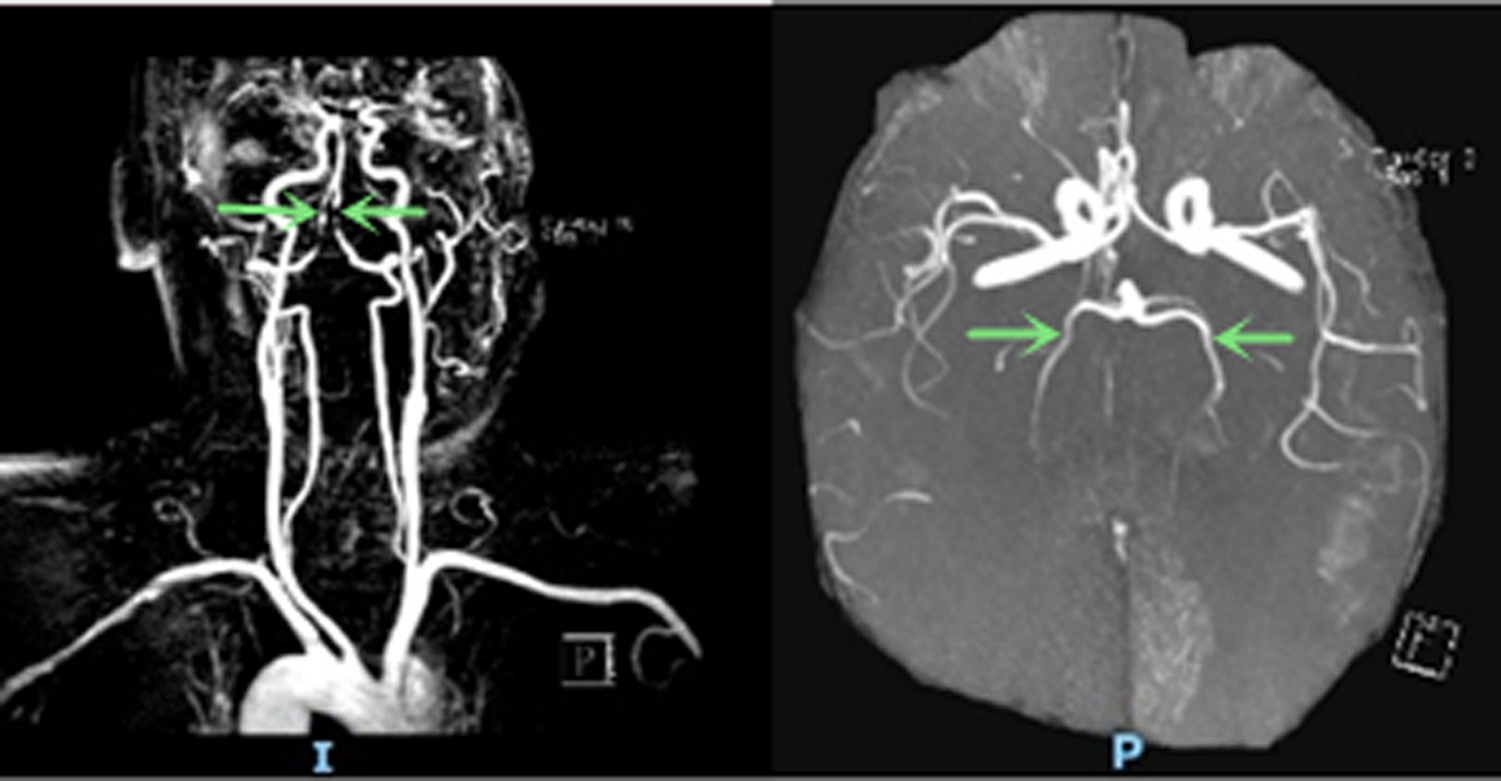
Repeat head and neck MRA (25 days after symptom onset) showed persistent stenosis in the bilateral V4 segments of the vertebral arteries and patency in bilateral posterior cerebral arteries (arrow).

Head and neck CT angiography (CTA) was performed, which showed significant stenosis in the basilar artery, and occlusion in the V4 segment of the left vertebral artery (Figure 2). Digital subtraction angiography (DSA) showed significant stenosis in the V4 segment of the right vertebral artery, occlusion in the V4 segment of the left vertebral, and left posterior cerebral arteries (Figure 3). Thrombectomy was initially considered, but it was aborted due to hemodynamic instability. CT brain the next day showed acute infarcts in the left parieto-occipital region and the left thalamus (Figure 4). Repeat magnetic resonance angiography (MRA) head and neck was performed 25 days after presentation, which showed persistent stenosis in the V4 segments of the bilateral vertebral arteries, but patent bilateral posterior cerebral arteries (Figure 5).

He had a normal hepatorenal function, interleukin-6, fibrinogen, urine analysis, erythrocyte sedimentation rate, and coagulation testing. He was discharged to an acute rehabilitation facility. Unfortunately, at a one-year clinical follow-up, he had persistent neurologic deficits. He required percutaneous endoscopic gastrostomy due to dysphasia and tracheostomy. He was also unable to ambulate independently.


**Ethical approval:** The research related to human use has been complied with all the relevant national regulations, institutional policies and in accordance with the tenets of the Helsinki Declaration, and has been approved by the authors’ institutional review board or equivalent committee.
**Informed consent:** Informed consent has been obtained from all individuals included in this study.

## 3 Discussion

Adverse reactions to bee or wasp stings may present with neurological, cardiovascular, renal, pulmonary, and ocular symptoms [1,2]. Stings associated with cerebral infarction are extremely rare, and the underlying pathological mechanisms remain unclear. Furthermore, there are no guidelines for the management of stroke secondary to bee stings. We list documented cases of cerebral infarctions after bee or wasp stings (Table 1). Among all the reports, the stroke symptoms presented over a wide range of times after sting, from 15 min to 4–8 days and up to 14 days [2,3,11,13]. There were more males than females in the reviewed literature, with a ratio of 4.2:1. The ages of patients ranged from 34 months old to 71 years old [3,9]. Most individuals did not have classical stroke risk factors. Only two documented cases had a history of hypertension and smoking [10,21]. Given the rarity of this phenomenon, the TOAST classification of our case may be best described as “stroke of other determined etiology.” We believe that bee venom caused multifocal vasospasm involving different arteries. This likely resulted in the pattern of ischemic stroke confirmed on imaging. Alternatively, there could have been thrombus formation in the right V4 segment that subsequently embolized to the left posterior cerebral artery (PCA). Given the presentation with headache and vasoconstrictive trigger, a diagnosis of RCVS could also be considered [28]. The RCVS2 score for our case was 3. The features supportive of RCVS is a vasoconstrictive trigger, while the features inconsistent with RCVS is not thunderclap headache. Another possibility is secondary infarction related to rt-PA [29]. However, we believe this is less likely.

Most patients had favorable outcomes with immediate treatment; however, some had poor outcomes and four patients died [2,7,22,24]. The natural history of stroke following bee or wasp stings was, therefore, varied in the available literature. The youngest patient who died was 30 years old, and the mean age of death amongst cases was 41 years [7]. The locations of stings mainly involved the head, face, and neck. One case reported a near-global distribution of stings to the patient’s body; it was associated with bilateral hemorrhagic cerebellar infarctions [7]. Principal neurological manifestations following the sting included facial weakness, hemiplegia, slurred speech, seizures, involuntary movements, and coma [2,19,24]. Renal failure, acute coronary syndrome, and arrhythmias were also reported in some cases [1,14]. Two cases developed hypotension, and one had hypotensive syncope [6,18,20]. Thirteen patients underwent cerebral angiography, of which nine were found to have large artery stenosis or occlusion. Six cases were found to have bilateral infarctions [7,12,13,17,19,27]. Three cases were found to have bilateral large artery stenosis or occlusion [5,21]. Four cases had combined intracerebral or subarachnoid hemorrhage [2,7,10,15,24]. Currently, there is no evidence to suggest a particular vascular territory is more susceptible. In addition to stroke, bee stings have been associated with other neurologic signs and symptoms, such as trigeminal neuralgia [30] and Parkinsonism [31]. Future research is needed to better understand the relationship between stings and neurologic sequelae.

There are no specific guidelines or expert consensus on treating cerebral infarction associated with bee or wasp stings due to limited reported cases and variations in presentations. According to reported cases, it is reasonable to consider treatment with epinephrine, methylprednisolone, antihistamine, and other suitable anti-allergic drugs at an early stage. Bees generally leave their stinging apparatuses in patients’ lesions after envenomation; the Vespidae attached to the sting site often persistently inject venom. Prompt removal of such stinging apparatuses is likely beneficial [17]. Two patients obtained favorable outcomes after intravenous rt-PA. Prompt administration of intravenous rt-PA should be considered if there are no contraindications [14,21]. It is also essential to promptly correct hypotension or insufficient perfusion [6,18]. Attempts of mechanical thrombectomy have not yet been reported; our case abandoned this procedure due to hemodynamic instability. However, mechanical thrombectomy may be considered if affected individuals have large vessel occlusions and no contraindications. The possible clinical benefits require further validation alongside attention to detail concerning related comorbidities and complications. For certain patients in whom the mechanism is consistent with vasospasm, intra-arterial vasodilators are another consideration.

To date, the pathophysiological mechanisms of bee or wasp sting-associated cerebral infarction are not fully elucidated. Wasps are members of the order Hymenoptera, suborder apocrita. Sensitization to wasp venom requires only a few stings. Also, symptoms may occur after a single string [27], including a variety of reactions related to neurological, renal, pulmonary, ocular, muscular, and cardiovascular systems [1].

Postulated mechanisms include the following:

1) Immune system hyper-functionality: Riggs et al. [5] reported a patient who experienced a wasp sting 14 years prior to presentation. Upon being stung a second time, severe allergic reactions and bilateral occlusion of the internal carotid arteries were reported. Riggs et al. considered a possible mechanism of immune system hyper-functionality.
2) Global cerebral hypoperfusion: Hypotension post-sting may be attributed to histamine and prostaglandin-2 induction [19]. Vidhate et al. [13] reported an 8-year-old boy with no abnormalities of the cerebral vasculature. However, he suffered symmetrical watershed infarction bilaterally. Additionally, two patients [6,18] were also reported to have anaphylaxis-induced cerebral infarction related to hypotension and likely cerebral hypoperfusion.
3) Retrograde stimulation of the superior cervical ganglion: [4,5] Riggs argued that venomous insect stings may lead to systemic immune responses and increased endothelial permeability of the distal ICA. Multiple ipsilateral facial or neck wasp stings can stimulate sympathetic innervation to the distal ICA via the superior cervical ganglion.
4) Disseminated intravascular coagulation (DIC): Jain [17] proposed that hemolysis and endothelial damage via toxins in honeybee venom contributed to DIC development resulting in the occlusion of blood vessels by widespread fibrin thrombi in the microcirculation.
5) Vasoconstriction: Bee venom contains vasoactive peptides such as thromboxane, leukotrienes, and other vasoactive mediators, causing vasoconstriction that can lead to ischemic stroke [10,21]. The patient we reported had previous MRA three years prior that was negative for intracranial vessel stenosis or occlusion. Furthermore, the PCA stenosis resolved on repeat MRA 25-days after the initial presentation. This suggests vasoconstriction may be a possible mechanism underlying cerebral infarction in our patient.

## 4 Conclusion

In summary, cerebral infarction following bee stings is rare. Various mechanisms have been proposed but there is no consensus on the most common pathophysiology or management approach. Further experiments, including in animal models, would be of great interest to elucidate the cellular responses to the venom that lead to vascular compromise and cerebral ischemia.
